# Comparison of intramedullary and extramedullary fixation of stable intertrochanteric fractures in the elderly: a prospective randomised controlled trial exploring hidden perioperative blood loss

**DOI:** 10.1186/s12891-016-1333-z

**Published:** 2016-11-15

**Authors:** Leyi Cai, Te Wang, Lu Di, Wei Hu, Jianshun Wang

**Affiliations:** Department of Orthopaedics Surgery, The Second Affiliated Hospital of Wenzhou Medical University, NO.109, XueYuan West Road, Luheng District, Wenzhou, Zhejiang Province 325000 People’s Republic of China

**Keywords:** Blood loss, Elderly, Extramedullary fixation, Intertrochanteric fracture, Intramedullary fixation

## Abstract

**Background:**

Hip fracture is a severe and common injury that occurs predominantly in the elderly. Blood loss in the perioperative period is associated with a greater risk of dying in anaemic patients. The aim of the study was to explore the best way to treat stable intertrochanteric fractures, taking hidden blood loss into account.

**Methods:**

This prospective, randomised blinded study included patients aged over 65 years with stable intertrochanteric fractures (Evans grades I and II). The patients were allocated to one of two groups treated via extramedullary or intramedullary fixation. Patient data were retrieved from electronic charts. Functional recovery was evaluated using the Functional Recovery Score of Zuckerman. Postoperative complications were also recorded. The formula of Nadler and Gross was used to calculate blood loss.

**Results:**

There were 92 patients in the extramedullary and 106 in the intramedullary group. Age, sex, the cause of injury, the type of fracture, the observed blood loss, functional recovery, time to union, complications, and American Society of Anesthesiologists classification did not differ significantly between the two groups (all *p*-values > 0.05). The frequencies of lung infection, electrolyte imbalance, and hypoproteinemia differed between groups (all *p*-values < 0.05). Total and hidden blood loss were higher in the intramedullary group (*p* = 0.001).

**Conclusion:**

Extramedullary (compared with intramedullary) fixation of stable intertrochanteric fractures significantly reduces perioperative blood loss but affords similar functional outcomes and times to union. In view of the morbidity and complications associated with acute anaemia and transfusions, extramedullary fixation may be the optimal choice for treatment of stable fractures, being associated with reduced blood loss.

**Trial registration:**

The study was retrospectively registered at the Chinese Clinical Trial Registry, number: ChiCTR-INQ-16009754, trial registration date: 6th Nov. 2016.

## Background

Intertrochanteric fracture is a severe and common injury that occurs predominantly in the elderly [1–4] and is associated with high rates of morbidity and mortality [5–7]. As populations age, the number of hip fractures increase; the mortality rates range from 15 to 30% [8]. In 2040, an estimated 512,000 hip fractures will occur in the United States alone, costing 16 billion dollars (USD) [5].

The optimal implant for repair of intertrochanteric fractures remains controversial. The options include extramedullary and intramedullary fixation. Dynamic hip screw (DHS) fixation is widely used to surgically treat intertrochanteric femoral fractures via extramedullary fixation. This device is considered to be the gold standard for management of such fractures in the elderly [9]. However, the DHS often fails to yield good results when used to treat unstable and reverse/oblique fractures, limiting the clinical utility thereof [10, 11]. Methods of intramedullary fixation include gamma nail placement and proximal femoral nail antirotation (PFNA). Several randomised controlled trials concluded that intramedullary fixation benefited patients with unstable peritrochanteric fractures, being associated with less blood loss and fewer complications than DHS placement [12, 13]. However, stable intertrochanteric fractures have not been studied in this context.

Hidden blood loss often occurs after intramedullary nailing of intertrochanteric fractures. Foss [14] reported that intramedullary nailing of hip fractures caused more hidden blood loss than did other forms of fixation, and arthroplasty. Patients with underlying anaemia are at a greater risk of dying in the perioperative period than are others [15, 16].

In many patients, and particularly in the elderly, functional recovery and safety during the perioperative period are more important than implant selection. The purpose of the present study was to define the best surgical treatment for stable intertrochanteric fractures, taking hidden blood loss into account.

## Methods

### Study population and design

Patients with stable comminuted intertrochanteric femoral fractures (Evans type I or II) [17] were randomised to a comparison of two different treatment methods in the trauma centre of our hospital from 2011 to 2014. The fourth author of this report, who was not involved in clinical treatment, tossed a coin to assign patients to one of the two groups (extramedullary or intramedullary fixation). The three senior surgeons on our team, all of whom have more than 15 years of clinical experience in treating intertrochanteric fractures, were familiar with both techniques. The surgeons (numbered from 1 to 3) were assigned, in turn, to perform all operations (extramedullary or intramedullary fixation). Thus, each surgeon performed similar numbers of both types of operations, minimising surgical bias.

We examined patient records and those of radiological and functional follow-up examinations. The inclusion criteria were age over 65 years; the ability to walk independently (with or without an aid) prior to fracture; and sustainment of a low-energy injury within 24 h prior to admission. The exclusion criteria were as follows: a compound femoral fracture, age under 65 years, a history of previous femoral fracture, any contraindication to surgery, nonambulatory status prior to the presenting injury, or any other traumatic fracture.

All patients agreed to participate in the trial; written informed consent was obtained. The study was approved by the Ethical Board of the Second Affiliated Hospital of Wenzhou Medical University (Zhejiang, China) and was performed in accordance with the ethical standards of the Declaration of Helsinki (1964).

### Data collection

The hospital records contained data on sex, age, height, weight, type of fracture, duration of operation, the observed blood loss during the operation, preoperative and postoperative hematocrit (Hct), and laboratory data (including the preoperative and postoperative hemoglobin (HB) and albumin levels). Some of the urea and creatinine levels had to be converted (by us) to mg/dL. The American Society of Anesthesiologists (ASA) scores were defined by a staff anaesthetist blinded to the treatment groups.

### Blood loss calculation

Total blood loss was calculated from the change in the Hct level and estimated total blood volume (TBV) derived using Nadler’s formula [18] (which considers sex), as follows:

$$ Females:\  blood\  volume\ (L) = height\ {(m)}^3\times 0.3561 + weight\ (kg)\times 0.03308 + 0.1833; $$

$$ Males:\  blood\  volume\ (L) = height\ {(m)}^3\times \kern0.62em  03669 + weight\ (kg)\kern0.5em \times \kern0.5em  0.03219 + 0.6041; $$

$$ Total\  red\  blood\  cell\  loss\ (L) = TBV\  preop.\kern0.5em \times \kern0.5em \left(Hct\  preop. - Hct\  postop.\right); $$

$$ Total\  blood\  loss\ (L) = Red\  blood\  cell\  loss/Hct\  preop. + volume\  of\  blood\  transfusions\  given\  intraoperatively; $$

$$ Hidden\  blood\  loss = Total\  blood\  loss - Observed\  blood\  loss. $$

Perioperative blood loss was calculated using the above formula and that of Gross [19]. The observed blood loss corresponds to the amount of liquid in the suction bottle minus the amount of liquid used to flush the wound and the net weight in gauze, gauze pads, and surgical towels. Laboratory tests were performed preoperatively and 1-day postoperatively in the same laboratory. The criteria for blood transfusion were an Hb level < 70 g/L or a level < 80 g/L when signs/symptoms of anaemia were present. The anaesthesia team, who were blinded to the type of fixation device used, managed fluid and electrolyte balance and blood transfusion.

### Clinical evaluation

The Functional Recovery Score (FRS) questionnaire of Zuckerman et al. [20] was used to measure functional recovery 6 and 12 months after operation. This was performed by a staff physical therapist blinded to treatment allocation, who calculated initial scores on admission and later administered the instrument again (twice) by telephone. The Zuckerman questionnaire contains 11 items and yields a score of 0–44; higher scores denote better functional capacity. Postoperative complications were assessed and recorded by another author blinded to the end of the study; wound infection, pneumonia, urinary tract infection, electrolyte imbalance, and hypoproteinemia were noted.

### Radiological evaluation

Anteroposterior and lateral hip radiographs were obtained immediately after operation to evaluate the quality of reduction and fixation of the fracture. Physical examination was performed, and lateral and anteroposterior radiographs of the hip were taken at each follow-up. Hardware was not routinely removed.

During follow-up, the time to union was assessed. Radiographic fracture union was defined as recanalisation of the trabeculae or a bridging callus visible on both radiographic views; delayed union was defined as no sign of fracture healing after 6 months; nonunion was defined as the absence of bone union after 9 months.

### Statistical analysis

Descriptive statistics were used to compare the basic characteristics of the two groups. Data were analysed using Student’s unpaired test and the chi-squared test. A *p*-value < 0.05 was taken to indicate statistical significance. Data are given as mean ± standard deviation. Statistical analyses were performed using SPSS for Windows software (ver. 19; SPSS Inc., Chicago, IL, USA).

## Result

**Fig. 1:**
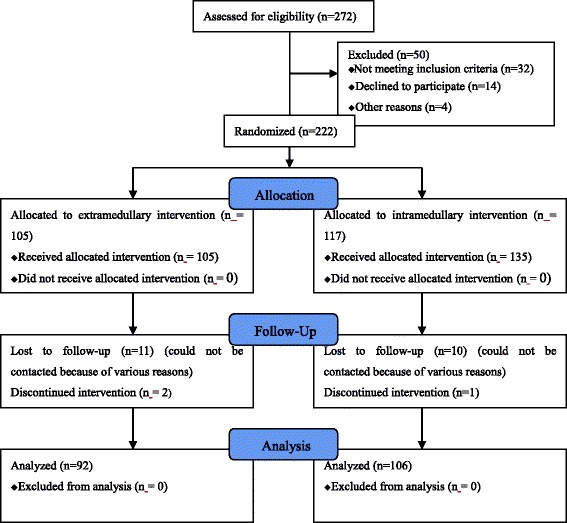
Consolidated Standards of Reporting Trials (CONSORT) 2010 flow diagram depicting fracture allotment in both groups

A total of 272 adult patients who suffered stable intertrochanteric fracture was assessed (Fig. 1). Thirty-two patients did not meet the inclusion criteria. A total of 18 patients met the inclusion criteria but were eventually excluded. Of these, 4 patients were excluded due to severe medical ailments, and the other 14 declined to accept the assigned treatment. Therefore, 222 patients, including 82 males and 140 females, of mean age 75.9 years (range, 65–100 years), met the inclusion criteria and participated in the study. Of these, a total of 24 were lost to follow-up for various reasons. The remaining 198 (68 males and 130 females) were followed-up for an average of 14 months (range, 12–16 months).

The extramedullary and intramedullary fixation groups were similar. Ninety-two patients were treated via extramedullary fixation, 106 via intramedullary fixation. The extramedullary fixation group contained 29 males and 63 females of mean age 75.9 years (range, 65–88 years). According to the Evans classification, there were 32 Type-I fractures and 60 Type-II fractures. There were 55 right- and 37 left-side hip injuries. The injury mechanisms included tumble accidents (77 fractures), traffic accidents (10 fractures), and other causes (5 fractures). The average time from initial injury to operation was 3.61 days; the average operative time was 44.7 min. The intramedullary fixation group consisted of 39 males and 67 females of mean age 75.9 years (range, 65–100 years). According to the Evans classification, there were 30 Type-I fractures and 76 Type-II fractures. There were 56 right- and 50 left-side hip injuries. The injury mechanisms included tumble accidents (80 fractures), traffic accidents (13 fractures), and other causes (13 fractures). The average time from initial injury to operation was 3.58 days; the average operative time was 46.3 min (Table 1). There were no significant differences between the two groups in any of sex, age, fracture classification, side of injured hip, injury mechanism, average time from initial injury to operation, average operative time, or ASA classification.

**Table 1:** Comparison of the general characteristics of the two groups

General information	Extramedullary group	Intramedullary group	*P* value
	*N* = 92	*N* = 106	
Age (yr)	75.9 ± 6.06	75.8 ± 6.20	0.954
Sex			
Male	29	39	
Female	63	67	
Evans classification			0.327
Type-I	32	30	
Type-II	60	76	
Side of injured			0.326
Right	55	56	
Left	37	50	
Injury mechanism			0.220
Fall from a stand height	77	80	
Traffic accident	10	13	
Other causes	5	13	
Time to operation (days)	3.61 ± 1.73	3.58 ± 1.57	0.89
Operative time (min)	44.7 ± 10.2	46.3 ± 8.61	0.24
ASA classification			
ASA I	2	3	
ASA II	50	53	0.814
ASA III	40	50	

The blood loss data are shown in Table 2. There was no significant between-group difference in any of weight, height, Hct preop, HB preop, or observed blood loss. However, the total and hidden blood losses in the extramedullary fixation group were lower than those in the intramedullary fixation group (both p-values = 0.001). The hidden blood loss was more than the observed blood loss in both groups (528.37 ± 386.91 mL versus 135.54 ± 36.48 mL and 720.51 ± 408.91 mL vs. 138.92 ± 37.69 mL for extramedullary and intramedullary fixation groups, respectively). Eight patients in the extramedullary group and 20 in the intramedullary fixation group required intra-operative blood transfusions due to low blood pressure. Four patients in the extramedullary and 10 in the intramedullary fixation group received blood transfusions in the first day after operation due to low Hb levels. The blood transfusion rate was 13.04% (12/92) in the extramedullary fixation group, significantly lower than 28.30% (30/106) in the intramedullary fixation group (*p* = 0.022; Table 2).

**Table 2:** Comparison the two groups of blood loss

Parameters	Extramedullary fixation	Intramedullary fixation	T or *X* ^2^ value	*P* value
Sample number (n)	92	106	—	—
Weight(kg)	60.56 ± 5.82	59.49 ± 5.56	1.33	0.1
Height(m)	1.64 ± 0.73	1.64 ± 0.7	0.07	0.95
Hematocrit, preop	0.326 ± 0.053	0.316 ± 0.049	1.45	0.15
Hemoglobin, preop (g/dl)	107.87 ± 18.37	104.82 ± 16.78	1.2	0.22
Total blood loss(ml)	663.91 ± 389.32	859.43 ± 411.07	3.42	0.001*
Observed blood loss(ml)	135.54 ± 36.48	138.92 ± 37.69	0.64	0.5
Hidden blood loss(ml)	528.37 ± 386.91	720.51 ± 408.91	3.38	0.001*
Blood transfusion rate	12(13.04%)	30(28.30%)	5.232	0.022*

*Significant value

Table 3 compares the FRSs and times to union in the two groups. In the extramedullary fixation group, the FRSs at baseline (pre-operatively) and at 6 and 12 months after surgery were 40.64 ± 2.47, 33.78 ± 3.04, and 35.96 ± 1.99, respectively, similar to those of the intramedullary fixation group (scores: 40.43 ± 2.72, 34.25 ± 2.91, and 36.10 ± 2.38; *p* = 0.577, *p* = 0.26, and *p* = 0.64, respectively). The median scores of the two groups were lower at 6 and 12 months post-operatively than pre-operatively, but started to improve at 6 months. The time to union did not differ significantly between the extramedullary and intramedullary fixation groups (13.29 ± 1.22 vs. 12.18 ± 1.30 weeks, respectively, *p* = 0.526).

**Table 3:** Comparison the two groups of function recovery scores and time to union

	*n*	Zuckerman FRC			Time to union(w)
		Pre-operative	6 months	12 months	
Extramedullary fixation	92	40.64 ± 2.47	33.78 ± 3.04	35.96 ± 1.99	13.29 ± 1.22
Intramedullary fixation	106	40.43 ± 2.72	34.25 ± 2.91	36.10 ± 2.38	12.18 ± 1.30
Test value		0.558	1.11	0.473	0.635
*P* value		*p* = 0.577	*p* = 0.26	*p* = 0.64	*p* = 0.526

*FRC* functional recovery scores

In terms of surgical and fracture-healing complications during follow-up, 5.4% (5/92) of the extramedullary fixation group developed lung infections, 10.87% (10/92) electrolyte imbalances, and 11.96% (11/92) hypoproteinemia; the figures for the intramedullary fixation group were 14.2% (15/106), 24.53% (26/106), and 25.47% (27/106), respectively; the between-group differences were significant (all *p*-values < 0.05; Table 4). No deep infection, delayed union, or nonunion was evident in either group. Both groups exhibited similar rates of superficial wound infection, urinary tract infection, mortality, and cutting of the lag screw (all *p*-values > 0.05; Table 4). One patient in the extramedullary fixation group suffered an implant failure; PFNA was used to heal the fracture. Four patients in the intramedullary and three in the extramedullary fixation group died during the second year of follow-up of causes unrelated to their operations.

**Table 4:** Postoperative complications of patients in the two groups

Parameters	Extramedullary fixation (*n* = 92)	Intramedullary fixation (*n* = 106)	*P* value
Superficial wound infection	4(4.3%)	3(2.8%)	0.42
Deep wound infection	0	0	—
Pneumonia	5(5.4%)	15(14.2%)	0.042*
Urinary tract infection	5(5.4%)	6(5.7%)	0.95
Mortality	3(3.2%)	4(3.8%)	0.58
Delayed union	0	0	—
Nonunion	0	0	—
Cutting of the lag screw	4(4.3%)	3(2.8%)	0.42
Implant failure	1(1.1%)	0	0.47
Electrolyte imbalance	10(10.87%)	26(24.53%)	0.013*
Hypoproteinemia	11(11.96%)	27(25.47%)	0.016*

The values are given as the *n*(%)

*P* values for between-group comparisons were determined by the chi-squared test and Fisher’s exact test for nominal variables

Statistically significant (*P* value < 0.05)

## Discussion

The optimal management of intertrochanteric fractures remains controversial. DHS and gamma nails have been most commonly used to fix such fractures over the last decade [21]. However, PFNA, the latest device, is considered to be near-perfect; intertrochanteric fractures are healed with minimal complications [10, 12]. Earlier randomised controlled trials and meta-analyses failed to reach unanimous conclusions. Lukas [22] performed a biomechanical in vitro study comparing DHSs and intramedullary nails for treatment of intertrochanteric fractures and found that extra- and intra-medullary osteosynthesis were comparable in terms of postoperative stiffness and survival during cyclic testing. As the nail failure load was significantly higher in the AO31-A2.3 fracture models tested, it was concluded that intramedullary implants should be preferred when fractures are unstable. Verettas [23], in a prospective randomised study, found no significant difference between the two methods of fracture stabilisation in terms of perioperative systemic effects. DHS placement is safe and effective, compared with novel intramedullary techniques, in already vulnerable patients with trochanteric fractures. We show that, in patients with stable fractures, intramedullary fixation did not afford any advantage.

In terms of functional recovery, Saudan et al. [24] measured social functioning and mobility 3, 6, and 12 months after surgery featuring PFNA or DHS placement. Although the intermediate findings were not given, no significant differences at the 1-year follow-up were evident, in terms of return to pre-fracture levels of ambulation and independence, between the PFN- and DHS-treated groups. Thus, it was concluded that intramedullary nails (such as the PFN) afforded no advantage over extramedullary devices (such as the DHS) in treatment of intertrochanteric fractures caused by low-energy trauma (Evans I and II grades). Another study [25] compared functional recovery 1 year after trochanteric nail- or DHS-mediated repair; final assessments were conducted over the telephone; no between-group difference in recovery score was noted. In our present study, we also found that there were no between-group differences either pre-operatively, or at 6 and 12 months, when intramedullary nails and extramedullary devices were placed. We thus suggest that both intramedullary and extramedullary approaches afford adequate fixation of stable intertrochanteric fractures. An uninjured, femoral lesser trochanter may be key in this context.

Perioperative blood loss has been associated with increased mortality, infection, deep venous thrombosis, renal and cardiac decompensation, and poorer functional results [15, 26, 27]; such blood loss should be considered when managing pertrochanteric fractures. Reduction of blood loss is one issue in such management; prevention of varus displacement, screw, and bone healing are further considerations. In our opinion, extramedullary fixation was associated with less blood loss than intramedullary fixation. Although the observed blood loss was similar in the two groups, the hidden blood loss differed significantly (*p* = 0.001) for the following reasons. First, the gluteus medius is crossed in the process of intramedullary fixation; this may damage the peritrochanteric arterial circuit. Second, insertion of an intramedullary device requires drilling of the greater trochanter, thus disturbing bone marrow neoangiogenesis [28]. In short, intramedullary fixation is associated with more hidden blood loss; this deserves more attention.

Several limitations of the study must be acknowledged. First, our results are based on rather inaccurate assessments of observed and calculated total blood losses. The precise calculation of blood loss depends on the accuracy of blood workups performed at admission and after surgery, and accurate measurement of intraoperative blood loss. Also, we did not explore possible confounders of blood loss. However, the formulae used have been applied in related works published in high-quality international journals, and are both reliable and practical. Second, our sample size was small; further studies with larger samples are required to confirm our findings.

We found that the perioperative incidences of pneumonia, hypoproteinemia, and electrolyte imbalance were higher in the intramedullary than the extramedullary fixation group, mainly due to a reduced perioperative blood volume. Although red cells were infused, total blood loss reduces the levels of other blood components, triggering hypoproteinemia, reduced immunity, lung infections, and other disorders.

Together, our data indicate that the final functional scores and the times to union were similar in both groups with stable fractures (Evans I and II grades). However, blood loss, specifically hidden blood loss, differed significantly between the groups.

## Conclusions

Extramedullary fixation (such as DHS placement) significantly reduces perioperative blood loss in patients with stable intertrochanteric fractures. Such fixation affords functional outcomes and times to union similar to those associated with intramedullary fixation. In view of the morbidity and complications associated with acute anaemia and transfusion, extramedullary fixation may be a good choice in such patients.

## Abbreviations

DHS: Dynamic hip screw
FRS: Functional Recovery Score
HB: Hemoglobin
Hct: Hematocrit
PFNA: Proximal femoral nail antirotation
TBV: Total blood volume
